# Pathobiological and Radiological Approach For Hepatocellular Carcinoma Subclassification

**DOI:** 10.1038/s41598-019-51303-9

**Published:** 2019-10-14

**Authors:** Francesco Vasuri, Matteo Renzulli, Silvia Fittipaldi, Stefano Brocchi, Alfredo Clemente, Salvatore Cappabianca, Luigi Bolondi, Rita Golfieri, Antonietta D’Errico

**Affiliations:** 1 Pathology Unit, S. Orsola University Hospital, Bologna, Italy; 2 Radiology Unit, Department of Diagnostic Medicine and Prevention, Sant’Orsola Hospital, University of Bologna, Bologna, Italy; 3 Radiology and Radiotherapy Unit, Department of Precision Medicine, University of Campania “L. Vanvitelli”, Naples, Italy; 4 Internal Medicine Unit, S. Orsola University Hospital, Bologna, Italy

**Keywords:** Hepatocellular carcinoma, Hepatocellular carcinoma

## Abstract

Many advances have been made in the imaging diagnosis and in the histopathological evaluation of HCC. However, the classic imaging and histopathological features of HCC are still inadequate to define patient’s prognosis. We aimed to find the link between new proposed morphovascular patterns of hepatocellular carcinoma (HCC) and magnetic resonance imaging (MRI) features to identify pre-operatory markers of biologically aggressive HCC. Thirty-nine liver nodules in 22 patients were consecutively identified. Histopathological analysis and immunohistochemistry for CD34 and Nestin were performed to identify the four different HCC morphovascular patterns. MRI was performed using gadolinium ethoxybenzyl diethylenetriamine pentaacetic acid. Three out of four morphovascular HCC patterns showed peculiar MRI features: in particular Pattern D (solid aggressive HCCs with CD34+/Nestin+ new-formed arteries) were isointense on T1-WI in 83% of cases and hyperintense on T2-WI in 50%. Five histologically-diagnosed HCC were diagnosed as non-malignant nodules on MRI due to their early vascularization and low aggressiveness (Pattern A). The comparison between histology and MRI confirms that a subclassification of HCC is possible in a pre-operatory setting. MRI seems to reinforce once more the identity of the different morphovascular HCC patterns and the possibility to pre-operatively identify HCCs with features of biological aggressiveness.

## Introduction

According to the most recent guidelines, the diagnosis and the first-line treatment of hepatocellular carcinoma (HCC) in cirrhotic patients is driven by imaging^1,2^. This is a quite peculiar case among solid tumors, which brought many advantages in the diagnosis and the treatment of HCC in patients who could not be biopsied, as well as in reducing the complications of biopsy (especially in cirrhotic patients) by limiting the indications for the biopsy itself^1^. Moreover, the real feasibility rate of liver biopsy in cirrhotic patients is not clear. The guidelines consider the cirrhotic patients as neoplastic and not neoplastic, with implications in the choice of therapy and in the correct allocation of patient in the liver waiting list for transplantation^1,2^. The two main limitations of this rough neoplastic stratification are: (1) where to collocate (and therefore how to treat) those cirrhotic patients with radiological diagnosis of High Grade Dysplastic Nodules (HGDN), early HCC (eHCC) and “undetermined” nodules, i.e. those nodules without typical radiological features of any of them^3^; (2) the absence of any biological and pathological differences among HCCs. Indeed, historically HCC has always been considered a “heterogeneous tumor”, but this explanation has not been defined or deepened. Edmondson’s grade and microvascular invasion (MVI) are the only accepted prognostic factors that the pathologist can provide, but only after surgery^1^. In fact, after biopsy Edmonson’s grade can be underestimated and MVI can be totally missed; moreover, MVI can be predicted by pre-treatment imaging^4^, but these data need to be prospectively confirmed. According to the diagnostic/therapeutic algorithm of most Guidelines, including the Italian ones^2^, the vast majority of the liver nodules are locally treated without histopatological analysis.

In the Barcelona Clinic Liver Cancer (BCLC) staging for HCC patients a wide prognostic range is observed, especially in group B (intermediate) patients^5^. For this reason, some authors tried to subclassify BCLC group B patients^6,7^, but none of the proposed subclassifications considered the biology and the pathology of HCCs, eHCCs and HGDNs.

A recent experience of the Pathology Unit of our Institution showed that it is possible to sort HCCs according to their morphovascular pattern, i.e. the tumor architecture and the maturity of intratumoral sinusoids and neoarteries^8^. Pattern A HCCs are well differentiated tumors, with lower incidence of MVI and parenchymal infiltration, as well as a lower expression of tumor growth factors and miRNAs. Conversely, Pattern D HCCs are solid high-grade tumors with few sinusoids and numerous neoarteries, more frequently arising from a non-cirrhotic liver^8,9^.

In our Istitution, also the radiological diagnosis of both HCC and precursor lesions such as eHCC and HGDN, was recently sharpened by applying a new diagnostic algorithm based on magnetic resonance imaging (MRI) with hepatospecific contrast agent^3^.

The aim of the present study is to find the link between the morphovascular HCC patterns and the corresponding imaging features, with the purpose to find a pre-operatory picture of those advanced HCC (of any dimensions) with biological and morphological features of aggressiveness. Our purpose is not to come back to perform the biopsy of every nodules in a cirrhotic background, but to identify, the tumor biological differences to correctly stratify the patients with HCC in the preoperative setting.

## Patients and Methods

### Case selection

The present study was approved in advance by the Ethical Committee of the S. Orsola University Hospital of Bologna (protocol code APHCC-2012, reference number 85/2013/O/Tess). All patients were treated according to the ethical guidelines of the 1975 Declaration of Helsinki (6^th^ revision, 2008); informed consent was obtained from each patient at the time of surgery.

We prospectively enrolled consecutive cirrhotic patients with HCC transplanted in 1 year (from Jun 2016 to May 2017). The inclusion criteria were: availability of clinical and MRI data performed no more than 6 months from liver transplantation (LT), together with nodule tissue for histopathological and immunohistochemical (IHC) analyses. Based on the available knowledge on mean HCC volume doubling time^10–12^, a six-month interval represents a reasonable choice for MRI until LT, since a longer interval can bias the comparison between radiological and histological data.

We excluded patients with previous locoregional treatments (percutaneous treatments, transarterial chemoembolization, etc.) and without naïve nodules.

### MRI techniques and image analysis

In this study, MRI was performed following previously described protocols^3,13^ by using hepatospecific contrast media such as gadolinium ethoxybenzyl diethylenetriamine pentaacetic acid (EOB Primovist; Bayer Schering Pharma, Berlin, Germany). The images obtained by MRI were blindly reviewed by two board-certified radiologists with more than 10 years of experience in liver imaging and more than 10 years of specific experience in the use of hepatospecific contrast media in liver MRI. They were blinded each other and to any information regarding histopathological features. Discordant cases were discussed collegially.

The radiologist recorded the number of lesions, the longest diameter and the liver location of each nodule. For each nodule, the signal intensity (1 = hyperintense; 2 = isointense; 3 = hypointense) was collected in T1 (in-phase and out-phase) and T2-weighted images and in arterial, portal-venous and hepatobiliary phase images. The diffusion weighted image (DWI) signal intensity was collected as follows: 0 indicated that the lesion was not observed or was isointense (absence of restriction) and 1 that the lesion had some degree of hyperintensity from minimal perceptible hyperintensity to maximal hyperintensity similar to that of the spleen (presence of restriction).

The diagnosis of HCC was reached according to the European Association for the Study of the Liver (EASL) criteria for nodules >1 cm (hypervascularity in the arterial phase and washout of contrast media in the portal-venous/delayed phases)^1^. The diagnosis of atypical HCC, eHCC and HGDN were performed according to the new imaging hallmark recently demonstrated^3^.

All nodules not fulfilling these criteria were classified as Regenerative Nodules (RNs).

### Histopathology and immunohistochemistry

Tissue samples from the nodules were taken from the surgical LT specimen for routine histopathological analysis. From formalin-fixed and paraffin-embedded tissue blocks, 2-µm-thick sections were cut for Haematoxylin-Eosin and Reticulin stains, as well as for IHC. At histological analysis, regenerative nodules (RN) were diagnosed as hepatocellular nodules with no cytological atypia, low cell density, and regular architecture; low-grade dysplastic nodules (LGDN) as nodules with slightly increased cell density^14^. High-grade dysplastic nodules (HGDN) showed mild architectural and/or cytological atypia, an increased cell density, a progressive loss of portal tracts, and occasional unpaired arteries^15^. Finally, early HCC (eHCC) showed stromal invasion, increased nuclear/cytoplasmic ratio, trabecular architecture and/or acinar structures, several unpaired arteries and eventually fatty changes^16–18^. In definite HCC, tumor grade according to Edmondson, tumor architecture and growth were evaluated, together with the occurrence of microvascular invasion (MVI).

IHC for CD34 (mouse monoclonal, clone Q-BEnd-10) was automatically performed by means of Benchmark ULTRA^®^ immunostainer following the manufacturer’s instructions (Ventana/Roche, Ventana Medical Systems). Nestin immunostaining (mouse monoclonal, clone 10C2, dilution 1:400, heat-mediated antigen retrieval) was manually performed as previously described in the liver^8^ with NovoLink Polymer Detection Kit (Novocastra, Newcastle, UK). Slides were scanned and acquired using a Hamamatsu Nanozoomer 2.0 RS instrument and images were processed using the dedicated software NDP.view V2.3.1.

### Histopathological morphovascular pattern of HCC

Morphovascular patterns of HCC were assigned based on tumor architecture and IHC for CD34 and Nestin, as previously described^8^. Briefly:

- Pattern A HCCs are well differentiated tumors, with microtrabecular/acinar architecture and CD34-positive and Nestin-negative sinusoids.
- Pattern B HCCs are similar in architecture, but they show activation of sinusoids, which are CD34-positive and Nestin-positive.
- Pattern C HCCs are high-grade macrotrabecular tumors, with macrotrabecolae lined by CD34-positive and Nestin-positive endothelia.
- Pattern D HCCs are solid high-grade tumors with true neoplastic neoarteries.

**Figure 1:**
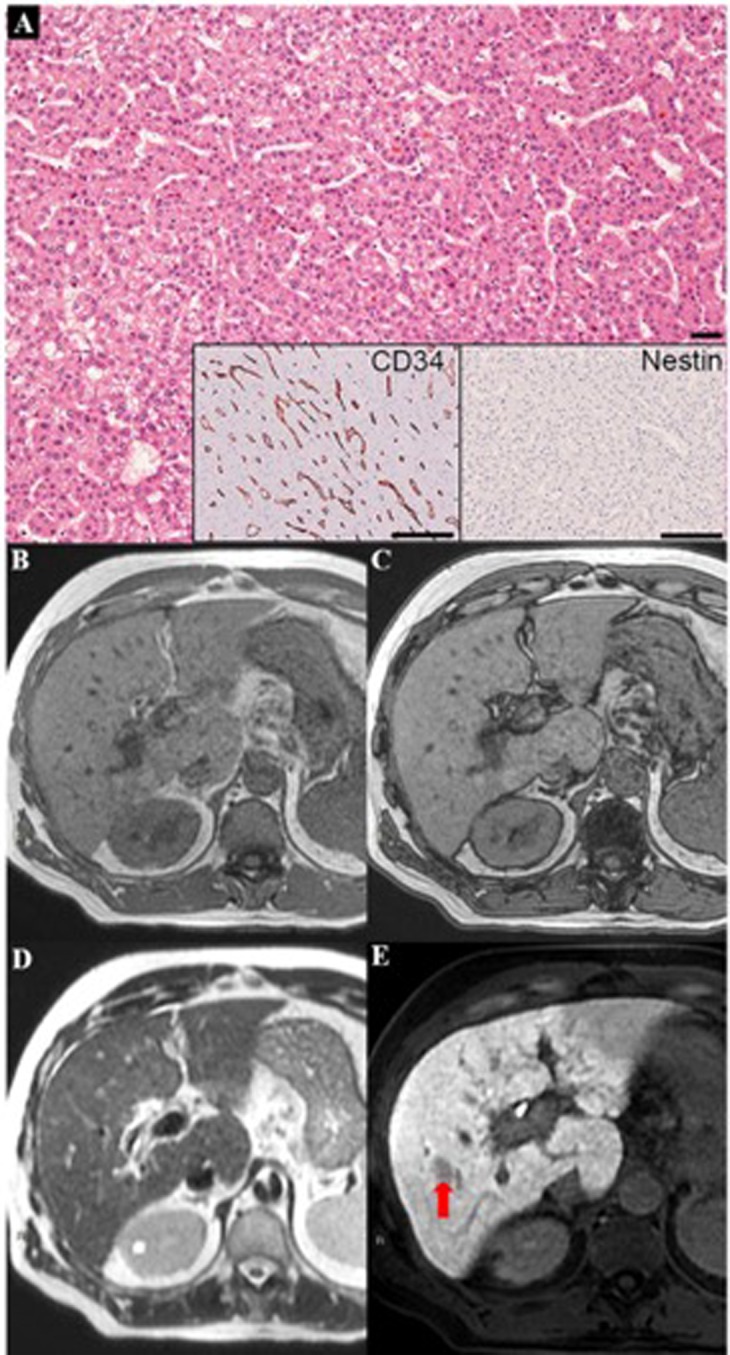
A case of HCC with morphovascular Pattern A, characterized by low tumor grade, prevalent microtrabecular architecture and, at immunohistochemistry, sinusoids positive for CD34 and negative for Nestin. (**A**) In MRI, a clear nodule was detectable in the liver segment VIII only in the hepatobiliary phase due to its well-defined hypointensity. (**E**) The same nodule did not demonstrate any imaging alterations in the basal sequences, such as T1-weighted images in-phase (**B**) and out-phase (**C**), and in T2-weighted image (**D**).

**Figure 2:**
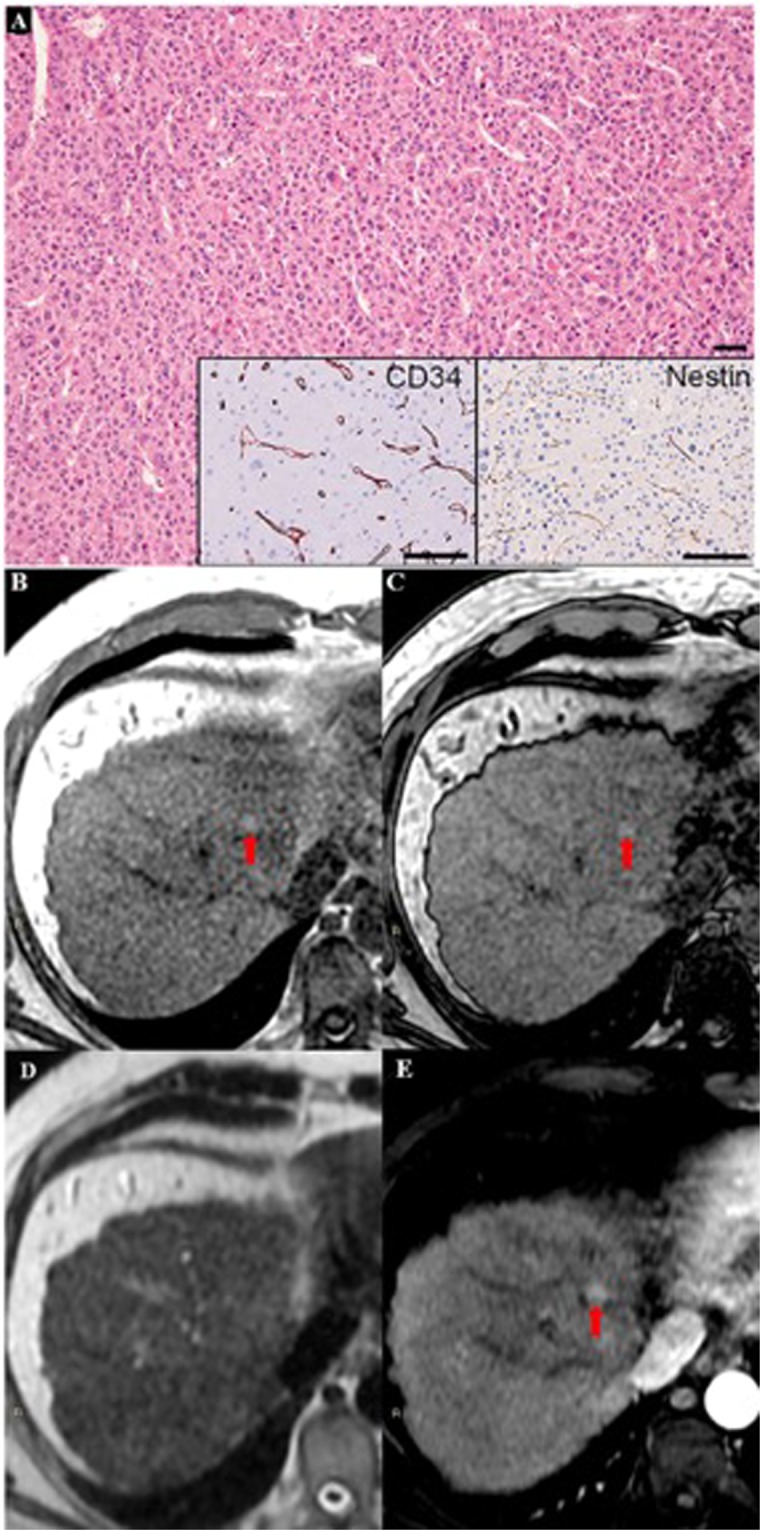
A case of HCC with morphovascular Pattern B, similar to the case in Fig. 1 in morphology, but with sinusoids positive for both CD34 and Nestin (**A**). In MRI, a clear nodule was identified in the liver segment IV, with hyperintensity in T1-weighted images in-phase (**B**) and out-phase (**C**), without hyperintensity in T2-weighted image (**D**) and with arterialization (**E**).

**Figure 3:**
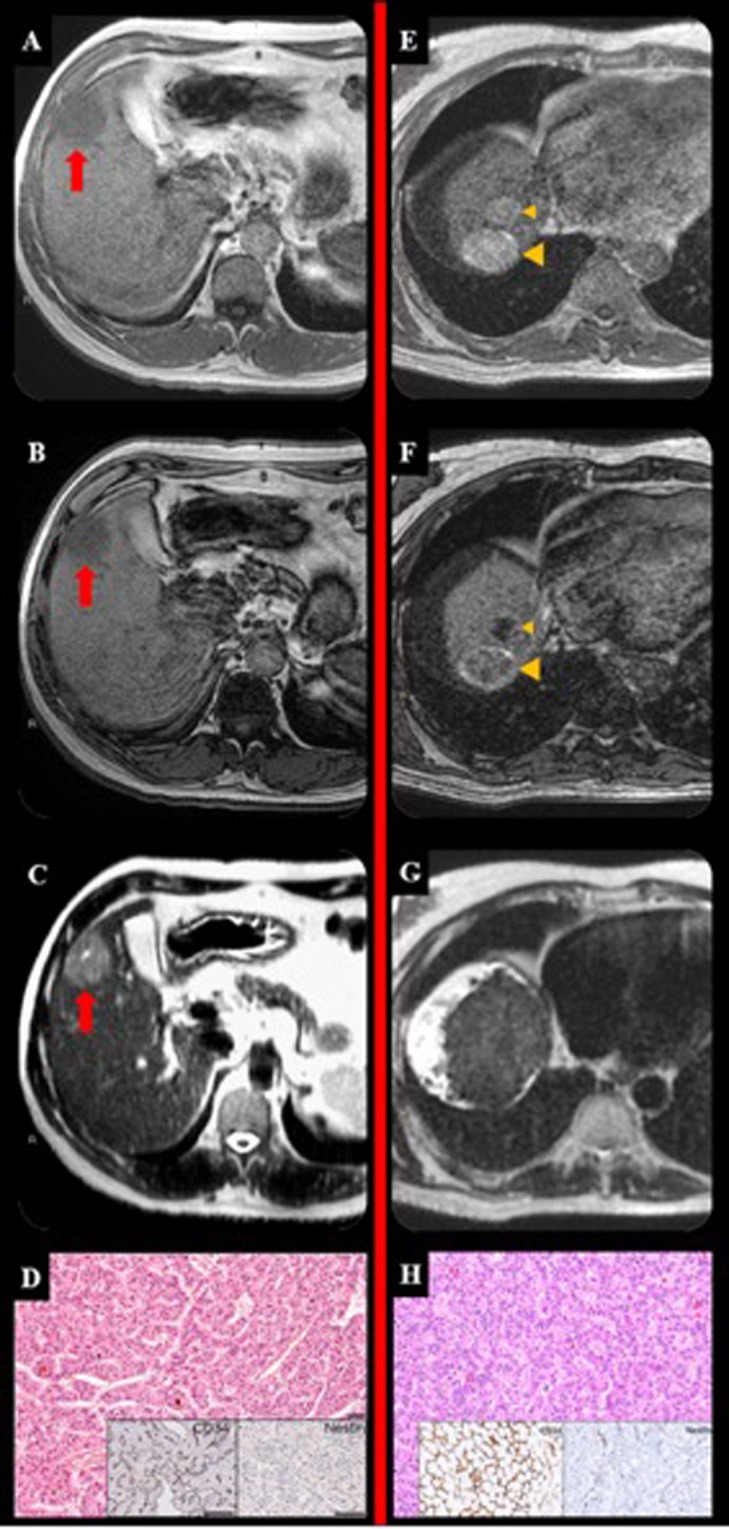
This figure illustrates two different cases of HCC with Pattern C (first patient: **A**–**D**; second patient: **E**–**H**). The HCC appeared different in all the basal sequences. In T1-weighted images in-phase the lesion appeared hypointense in A (arrow) and hyperintense in D (arrow-heads). In T1-weighted images out-phase the lesion appeared hypointense in B (arrow) and with different degree of signal drop-out in E (arrow-heads). In T2-weighted images the lesion appeared hyperintense in c (arrow) and isointense in D (arrow-heads). However, at histopathological analysis, they share the same macrotrabecular morphology and vascularization (**D**,**H**).

**Figure 4:**
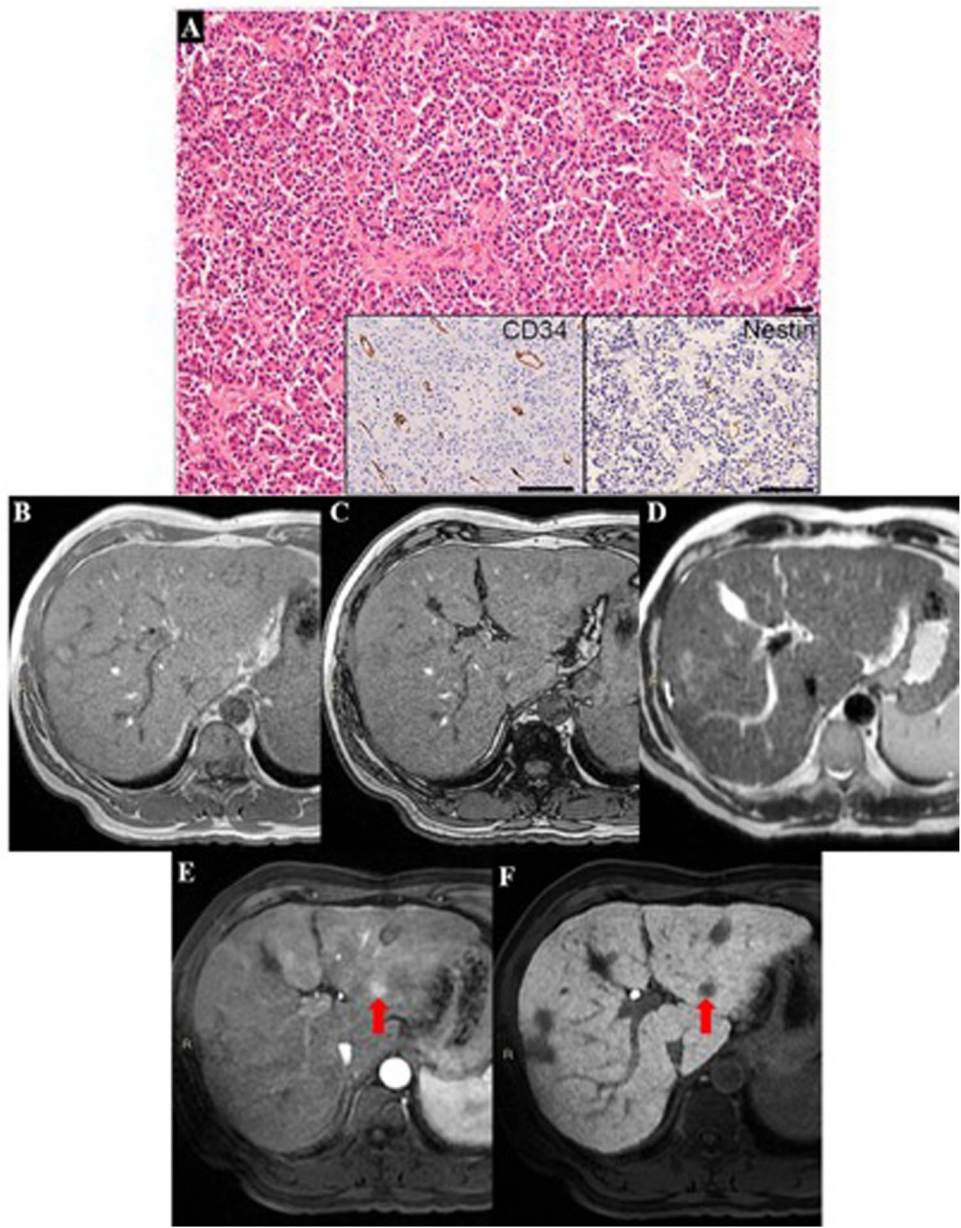
A case of HCC with morphovascular Pattern D, characterized by high tumor grade, prevalent solid architecture and CD34-positive Nestin-positive neoarteries. (**A**) In MRI, a clear nodule was detectable in the liver segment II with hyperintensity in the arterial phase (**E**) coupled with hypointensity in the hepatobiliary phase. (**F**) The same nodule did not demonstrate any imaging alterations in the basal sequences, such as T1-weighted images in-phase (**B**) and out-phase (**C**), and in T2-weighted image (**D**).

Figures 1–4 show examples of these 4 different morphovascular patterns.

### Statistical analysis

Statistical analysis was performed by means of SPSS^®^ software for Windows, ver. 20. Variables are reported as means ± standard deviations, ranges, and frequencies. The cross-correlations between discrete variables ─MRI features and histological features—were analyzed with the chi-squared test. A *p*-value less than or equal to 0.05 was considered statistically significant.

## Results

### Patients and selected nodules

Following all inclusion criteria, the radiologist finally identified 39 nodules from 22 patients, 18 (81.8%) males, mean age at surgery 56.1 ± 9.9 years (range 43–78 years). The mean number of evaluated nodules for patient was 1.8 ± 1.6: in particular, 14 patients had 1 suspicious nodule, 2 patients had 2 nodules, 2 patients had 3, 2 patients had 4, and 1 patient had 7 nodules. Mean nodule dimension was 1.9 ± 0.8 cm.

### Lesions diagnosis at MRI and histopathology

The MRI imaging features of the 39 nodules are listed in Supplementary Table 1. Based on these MRI parameters, the radiological diagnosis of the single nodules was: RN in 7 (17.9%) cases, HGDN in 5 (12.8%), eHCC in 8 (20.5%), and HCC in 19 (48.8%). Therefore 12 (30.8%) nodules were diagnosed as non-malignant lesions, and 27 (69.2%) nodules as malignant lesions.

The histopathological diagnosis of the single nodules was: RN in 6 (15.4%) cases, HGDN in 1 (2.6%), eHCC in 9 (23.1%), and overt HCC in 23 (58.9%). Therefore, 7 (17.9%) nodules were non-malignant lesions, and 32 (82.1%) were malignant lesions. Since morphovascular patterns classify the overt HCCs, the 7 non-HCC lesions were not assigned to any pattern.

After the morphological assessment of tumor architecture and IHC in the 32 histological HCCs, 15 (46.9%) HCC were classified as Pattern A, 6 (18.7%) HCC as Pattern B, 6 (18.7%) HCC as Pattern C, and 5 (15.6%) HCC as Pattern D.

Cross-analyzing the MRI and histopathological data, we observed that all benign nodules at histopathology were diagnosed as non-malignant at MRI as well (P < 0.001; Table 1). Indeed, the 7 non-malignant nodules received a radiological diagnosis of RN in 6 cases and HGDN in 1. Radiological eHCC were all confirmed as malignant at histology, with pattern A in 62.5% of cases.

**Table 1:** Cross-analysis between MRI and histopathological diagnoses (sorted by morphovascular pattern from A to D.

		Vascular Pattern					Total
		None	A	B	C	D	
MRI	RN	6	1	0	0	0	7
	HGDN	1	3	0	0	1*	5
	eHCC	0	5	0	2	1	8
	HCC	0	6	6	4	3	19
Total		7	15	6	6	5	39

*Discordant case (see text).

The five non-concordant nodules were histologically-diagnosed HCC, which received a MRI diagnosis of non-malignant nodules. Four out of these 5 cases were HCCs with a morphovascular Pattern A, diagnosed as HGDN in 3 cases and RN in one. IHC studies showed minimal endothelial activation. Moreover, the mean time lapse between MRI and resection in these cases was close to 6 months. The other discordant lesion was actually a HGDN at histopathology, albeit a focus of HCC with morphovascular Pattern D was identified in its dysplastic context. This case represents the only advanced HCC not diagnosed as malignant at MRI in our series, and was excluded from further analyses, since it represents an exception.

### Correlations among MRI and morphovascular patterns

Combining MRI features we can observe that HCCs with different morphovascular patterns showed distinctly imaging pictures. In particular:

- Pattern A HCCs (Fig. 1) showed isointensity in both T1in and T1out in 53.3% of cases, as well as isointensity in T2 in 66.7%. As stated above, at MRI 4 (26.7%) of them were diagnosed as non-neoplastic, 5 (35.7%) as eHCCs and 6 (42.9%) as HCCs.
- Pattern B HCCs (Fig. 2) showed hyperintensity in both T1in and T1out without hyperintensity in T2 4 out of 6 (66.7%) of cases: these characteristics are ascribable to the so-called glycogen nodule^19^.
- Pattern C HCCs (Fig. 3) showed the highest radiological variability in basal MRI, albeit all cases were hypointense in portal, late and HB phases.
- Pattern D HCCs (Fig. 4) were always isointense in T1in and T1out, and they were hyperintense in T2 in half cases. All cases were hypointense in portal, late and HB phases.

Table 2 reports the detailed cross-match among each MRI features, such as T1 (hyperintensity, isointensity, hypointensity) and the others, and each different morphovascular pattern. According this cross-analysis, the radiological variables significantly correlated with histopathological diagnosis and morphovascular pattern were: T1in (P = 0.034, chi-square test), T1out (P = 0.030), portal phase (P = 0.008), late phase (P = 0.001), HB phase (P < 0.001), and DWI (P = 0.015).

**Table 2:** MRI characteristics of the nodules analyzed, according to the morphovascular pattern.

		Morphovascular Pattern					Total	Sig. (chi-square)
		None	A	B	C	D		
T1 in	Hyper	6	6	4	3	0	19	
	Iso	1	8	2	1	4	16	**P = 0**.**034**
	Hypo	0	1	0	2	0	3	
Total		7	15	6	6	4	38	
T1 out	Hyper	0	3	4	2	0	9	
	Iso	7	8	2	2	4	23	**P = 0**.**030**
	Hypo	0	4	0	2	0	6	
Total		7	15	6	6	4	38	
T2	Hyper	1	5	2	3	2	13	
	Iso	6	10	4	2	2	24	n.s.
	Hypo	0	0	0	1	0	1	
Total		7	15	6	6	4	38	
ARTERIAL	Hyper	2	6	6	4	3	21	
	Iso	5	9	0	2	1	17	P = 0.056
Totale		7	15	6	6	4	38	
PORTAL	Hyper	0	0	1	0	0	1	
	Iso	6	4	3	0	0	13	**P = 0**.**008**
	Hypo	1	11	2	6	4	24	
Total		7	14	6	6	4	38	
LATE	Iso	6	3	0	0	0	9	
	Hypo	1	12	6	6	4	29	**P = 0**.**001**
Total		7	15	6	6	4	38	
HB	Iso	6	1	0	0	0	7	
	Hypo	1	14	6	6	4	31	**P < 0**.**001**
Totale		7	15	6	6	4	38	
DWI	NO	7	5	1	2	1	16	
	YES	0	10	5	4	3	22	**P = 0**.**015**
Totale		7	15	6	6	4	38	

## Discussion

The present paper copes with an old issue, the radiological and histopathological diagnosis of HCC, from a novel point of view. The diagnostic setting of HCC proceeded fast in the last decades but not so fast as for other human tumors, despite many innovations such as the hepatospecific contrast agent in MRI. In fact, Gd-EOB-DTPA was developed as hepatobiliary MRI agent back in 1992, and used to classify HCC. Already back in 1997, Reimer *et al*. demonstrated the usefulness of Gd-EOB-DTPA in the differential diagnosis of HCC with metastases and hemangiomas^20^. More recently, Joo *et al*. emphasized the value of Gd-EOB-DTPA for HCC diagnosis, and in particular highlighted that the signal intensity on hepatobiliary phase is an indicator of high tumor grade and high rate of recurrence after surgery^21^. Indeed, in the last guidelines on the HCC management, such as EASL 2018^1^ and the American Association for the Study of the Liver Disease (AASLD) 2018^22^, the radiological criteria for HCC diagnosis are quite similar to the previous versions starting from 2005^23^, despite new proposals and many results demonstrated by imaging^3,24,25^. Moreover, in the AASLD guidelines HCC precursor lesions such as HGDN are not even mentioned. As for the histopathological contributions, the diagnostic criteria for HCC remain the same as described by the International Consensus Group in 2009^14^, with some advances in the differential diagnosis between HGDN and eHCC thanks to IHC^26^. Nowadays, the different outcomes of HCC patients present in the literature, especially in intermediate stage of BCLC staging system^5^, are not justifiable nor predictable by the recent guidelines. Some efforts had been made in order to subclassify these patients, like the subclassification of BCLC stage B from Bolondi^6^, but the prognostic differences among published series was not overcame, probably due to the absence of a HCC morpho-biological characterization.

The purpose of our study was to describe different morphologies in HCC and to translate them at imaging, therefore in the pre-operatory setting. This new approach is promising, but the lack of an adequate comparison in the literature makes it difficult to discuss.

Our results start from a previously proposed histopathological subclassification of HCC, based on tumor architecture and vascular pattern^8^, as well as a new imaging flowchart from our Institution, that proposes diagnostic criteria not only for the radiological diagnosis of HCC, but also of pre-neoplastic nodules in patients under surveillance for HCC^3^.

The 4 different histopathological morphovascular HCC patterns express different growth factors and microRNAs^8,9^, and belong to different stages of HCC progression. To date, no data are available on prognosis, since the study groups are too heterogeneous. However, Pattern A HCC, more frequently found in cirrhosis, showed a lower incidence of infiltrative margins and MVI at histology^8^, the latter being a strong independent factor of poor prognosis in HCC patients^27^. The identification of Pattern A HCC is likely to define a group of HCC patients with a specific tumor prognosis compared to the other three groups, and this is particularly useful in the radiological pre-operative setting.

In our series, 73.3% of Pattern A HCCs were correctly identified as malignant. In particular, nearly half of these malignant nodules were radiologically diagnosed as eHCC. This is a further clue concerning the low biological aggressiveness of Pattern A HCCs compared to Pattern B, C and D. Moreover, 4 discordant cases were seen in this group, which represents the 80% of discordant cases (4 out of 5): only one was a true false negative case, since it was diagnosed as RN on MRI. The others were diagnosed as HGDN, a known pre-malignant lesions that usually evolves into malignant lesions^28^. Our radiological diagnosis could be justified by the absence of an overt vascularization, reflected by the absence of hyperintensity in the arterial phase of MRI and the absence of restriction in DWI. These radiological features correlate with the minimal endothelial activation found at histology. Our imaging approach allows the identification of pre-malignant lesions, as well as malignant lesions with low aggressiveness in 93.3% of cases. The paradox of these lesions is that their imaging diagnosis in cirrhotic livers is based on vascularization, HB phase and DWI, but more than half of them are not identifiable at basal imaging.

Two-third of Pattern B HCCs showed the imaging features of the so-called glycogen nodules, i.e. hyperintensity in both T1in and T1out without hyperintensity in T2^19^. This features allow the radiological distinction between Pattern B and Pattern A HCCs, reinforcing our hypothesis that, despite the similar architecture, they are different biological entities.

Pattern D HCCs were always isointense in T1in and T1out, but, differently from Pattern A, they were hyperintense in T2 in half cases. In non-cirrhotic patients under surveillance for HCC, the finding of a nodule with malignant features, not identifiable in basal imaging but hyperintense in T2, should raise the suspect of a Pattern D HCC. Pattern D HCCs show the most advanced histological neoangiogenesis^8^, and they are likely to represent the most advanced in the histological progression. Whether this corresponds to a higher tumor aggressiveness (and poorer prognosis) compared to the other patterns is still to be established. If this hypothesis would be confirmed, nodules with radiological profile of Pattern D should undergo biopsy.

One limitation of the present study was represented by the small number of nodules analyzed, and consequent weak statistical power. However, this small population is due to the stringent inclusion criteria, such as availability of the whole naïve nodule for histology, as well as MRI with hepato-specific contrast media performed no more than 6 months before surgery, as well as our new recent morphovascular classification of HCC, proposed in 2016. Another study limitation is the intrinsic qualitative nature of both MRI and histopathological analysis. Probably in the future the extensive use of radiomics might overcome this limitation. However, in the daily clinical practice, the application of guidelines is still necessary for the management of these qualitative medical disciplines. Surely, more standardized protocols and quantitative methods could improve the evaluation of MRI features in the next future, ensuring a greater reproducibility of this technique in the clinical practice and avoiding the bias of qualitative approach.

In conclusion, the comparison between histology and MRI with hepato-specific contrast media confirms that a subclassification of HCC is possible in pre-operatory setting. Indeed, MRI seems to reinforce once more the identity of the different morphovascular HCC patterns. Further studies are needed in order to validate our results, and to establish their prognostic implications.

## Supplementary information

Supplementary Table 1

## References

[1] European Association for the Study of the Liver (2018). EASL Clinical Practice Guidelines: Management of hepatocellular carcinoma. J Hepatol.

[2] Italian Association for the Study of the Liver (AISF) (2013). Position paper of the Italian Association for the Study of the Liver (AISF): the multidisciplinary clinical approach to hepatocellular carcinoma. Dig Liver Dis.

[3] Renzulli M (2018). New hallmark of hepatocellular carcinoma, early hepatocellular carcinoma and high-grade dysplastic nodules on Gd-EOB-DTPA MRI in patients with cirrhosis: a new diagnostic algorithm. Gut.

[4] Renzulli M (2016). Can current preoperative imaging be used to detect microvascular invasion of hepatocellular carcinoma?. Radiology.

[5] European Association For The Study of the Liver; European Organisation for Research and Treatment of Cancer. EASL-EORTC clinical practice guidelines: management of hepatocellular carcinoma. *J Hepatol* **56**, 908–943 (2012). 10.1016/j.jhep.2011.12.001 22424438

[6] Bolondi L (2012). Heterogeneity of patients with intermediate (BCLC B) Hepatocellular Carcinoma: proposal for a subclassification to facilitate treatment decisions. Semin Liver Dis.

[7] Yamakado K (2014). Prognosis of patients with intermediate-stage hepatocellular carcinomas based on the Child-Pugh score: subclassifying the intermediate stage (Barcelona Clinic Liver Cancer stage B). Jpn J Radiol.

[8] Vasuri F (2016). Facing the enigma of the vascular network in hepatocellular carcinomas in cirrhotic and non-cirrhotic livers. J Clin Pathol.

[9] Fittipaldi S (2017). miRNA Signature of Hepatocellular Carcinoma Vascularization: How the Controls Can Influence the Signature. Dig Dis Sci.

[10] Barbara L (1992). Natural history of small untreated hepatocellular carcinoma in cirrhosis: a multivariate analysis of prognostic factors of tumor growth rate and patient survival. Hepatology.

[11] Ebara M (1998). Natural course of small hepatocellular carcinoma with underlying cirrhosis. A study of 30 patients. Hepatogastroenterology.

[12] Sheu JC (1985). Growth rate of asymptomatic hepatocellular carcinoma and its clinical implications. Gastroenterology.

[13] Tovoli F (2018). Inter-operator variability and source of errors in tumour response assessment for hepatocellular carcinoma treated with sorafenib. Eur Radiol.

[14] International Consensus Group for Hepatocellular Neoplasia (2009). Pathologic diagnosis of early hepatocellular carcinoma: a report of the international consensus group for hepatocellular neoplasia. Hepatology.

[15] Watanabe S (1983). Morphologic studies of the liver cell dysplasia. Cancer.

[16] Kojiro M, Nakashima O (1999). Histopathologic evaluation of hepatocellular carcinoma with a special reference to small early stage tumor. Semin Liver Dis.

[17] Hytiroglou P, Park YN, Krinsky G, Theise ND (2007). Hepatic precancerous lesions and small hepatocellular carcinoma. Gasteroenterol Clin N Am.

[18] Roskams T, Kojiro M (2010). Pathology of early hepatocellular carcinoma: conventional and molecular diagnosis. Semin Liver Dis.

[19] Kim YS, Song JS, Lee HK, Han YM (2016). Hypovascular hypointense nodules on hepatobiliary phase without T2 hyperintensity on gadoxetic acid-enhanced MR images in patients with chronic liver disease: long-term outcomes and risk factors for hypervascular transformation. Eur Radiol.

[20] Reimer P (1997). Enhancement characteristics of liver metastases, hepatocellular carcinomas, and hemangiomas with Gd-EOB-DTPA: preliminary results with dynamic MR imaging. Eur Radiol.

[21] Joo I, Lee JM (2016). Recent Advances in the Imaging Diagnosis of Hepatocellular Carcinoma: Value of Gadoxetic Acid-Enhanced MRI. Liver Cancer.

[22] Heimbach JK (2018). AASLD guidelines for the treatment of hepatocellular carcinoma. Hepatology.

[23] Bruix J, Sherman M (2005). American Association for the Study of Liver Diseases. Management of hepatocellular carcinoma. Hepatology.

[24] Renzulli M, Golfieri R (2016). Proposal of a new diagnostic algorithm for hepatocellular carcinoma based on the Japanese guidelines but adapted to the Western world for patients under surveillance for chronic liver disease. J Gastroenterol Hepatol.

[25] Choi SH (2016). Diagnostic criteria for hepatocellular carcinoma ⩽3 cm with hepatocyte-specific contrast-enhanced magnetic resonance imaging. J Hepatol.

[26] Di Tommaso L (2007). Diagnostic value of HSP70, glypican 3, and glutamine synthetase in hepatocellular nodules in cirrhosis. Hepatology.

[27] Llovet JM, Schwartz M, Mazzaferro V (2005). Resection and liver transplantation for hepatocellular carcinoma. Semin Liver Dis.

[28] Borzio M (2003). Impact of large regenerative, low grade and high grade dysplastic nodules in hepatocellular carcinoma development. J Hepatol.

