# Supplementary material for: Pathobiological and Radiological Approach For Hepatocellular Carcinoma Subclassification
Source: Sci Rep. 2019 Oct 14;9:14749. doi: 10.1038/s41598-019-51303-9 (PMC6791846; doi:10.1038/s41598-019-51303-9)
Supplement: Supplementary file 1 — Supplementary Table 1 [file 41598_2019_51303_MOESM1_ESM.pdf]

PATHOBIOLOGICAL AND RADIOLOGICAL APPROACH FOR HEPATOCELLULAR CARCINOMA SUBCLASSIFICATION

Francesco Vasuri[1*], Matteo Renzulli[2*], Silvia Fittipaldi[1], Stefano Brocchi[2], Alfredo Clemente[3], Salvatore Cappabianca[4], Luigi Bolondi[4], Rita Golfieri[2§], Antonietta D'Errico[1§]

[1]Pathology Unit, S. Orsola University Hospital, Bologna, Italy.

[2]Radiology Unit, Department of Diagnostic Medicine and Prevention, Sant'Orsola Hospital, University of Bologna, Bologna, Italy.

[3]Radiology and Radiotherapy Unit, Department of Precision Medicine, University of Campania "L. Vanvitelli", Naples, Italy.

[4]Internal Medicine Unit, S. Orsola University Hospital, Bologna, Italy.

	Hypointensity	*Isointensity*	*Hyperintensity*
T1in	19 (48.7%)	16 (41.0%)	4 (10.3%)
Tiout	9 (23.1%)	23 (59.0%)	7 (17.9%)
T2	14 (35.9%)	24 (61.5%)	1 (2.6%)
Arterial Phase	21 (53.8%)	18 (46.2%)	0 (0.0%)
Portal Phase	1 (2.6%)	14 (35.9%)	24 (61.5%)
Late Phase	0 (0.0%)	10 (25.6%)	29 (74.4%)
Biliary Phase	0 (0.0%)	7 (17.9%)	32 (82.1%)
DWI	17 (43.6%)*		22 (56.4%)

Supplementary Table 1 MRI features (frequencies and percentages) of the 39 nodules analyzed.

*absence of restriction in DWI.
